# SIMLINK enables accurate variant pathogenicity prediction through modeling the gene-variant-feature association structure

**DOI:** 10.1093/bioinformatics/btag601

**Published:** 2026-08-10

**Authors:** Hong-Dong Li, Chenlu Wang, Dongfang Yan, Wenkui Huang, Zongxuan Li, Shaokai Wang

**Affiliations:** School of Computer Science and Engineering, Central South University, Changsha, Hunan 410083, P.R. China; Hunan Provincial Key Lab on Bioinformatics, Changsha, Hunan 410083, P.R. China; School of Computer Science and Engineering, Central South University, Changsha, Hunan 410083, P.R. China; School of Computer Science and Engineering, Central South University, Changsha, Hunan 410083, P.R. China; School of Computer Science and Engineering, Central South University, Changsha, Hunan 410083, P.R. China; School of Computer Science and Engineering, Central South University, Changsha, Hunan 410083, P.R. China; School of Computer Science and Engineering, Central South University, Changsha, Hunan 410083, P.R. China; Department of Mathematics, Hong Kong University of Science and Technology, Hong Kong, P.R. China

## Abstract

**Motivation:**

Predicting variant pathogenicity is crucial for clinical genetics. Existing approaches face two primary limitations. First, biologically, data for pathogenicity prediction often lacks explicit modeling of the gene-variant-feature association structure. A single gene can harbor multiple variants, and each variant can be characterized by multiple features intrinsically associated with its parent gene. Current methods fail to explicitly model the gene-variant-feature association, thus limiting their performance. Second, methodologically, the variant-pathogenicity association is often assumed to comprise a linear component alongside a nonlinear one. However, current methods typically do not explicitly model the linear component, often failing to disentangle the linear component that might be better addressed with a linear approach.

**Results:**

To overcome these limitations, we introduce simultaneous modeling of linear and nonlinear components of knowledge graph (SIMLINK). This novel approach leverages a knowledge graph to model gene-variant-feature associations and a linear model to isolate the linear component. We begin by constructing a variant-centered knowledge graph, comprising over 8 million triplets, which explicitly models the associations between genes, variants, and features. Subsequently, the linear and nonlinear components are learned using a combination of linear and graph neural networks. We train SIMLINK on ClinVar variants. Benchmarking experiments on independent test sets demonstrate its superior prediction on both missense and synonymous variants compared to state-of-the-art methods, including CADD and AlphaMissense. We evaluate the impact of allele frequencies on prediction performance. Applied to variants implicated in Autism Spectrum Disorder, SIMLINK effectively distinguished between high- and low-confidence variants, and critically, the genes harboring top-ranked variants are highly pathogenic.

**Availability and implementation:**

The source code is freely available at https://github.com/Chen-LuWang/SIMLINK.

## 1 Introduction

Single nucleotide variants (SNVs) are major contributors to complex diseases, including cancers and neurological diseases. The advent of next-generation sequencing (NGS) has led to the identification of millions of such variants, yet this rapid pace of discovery has created a significant interpretive challenge. For the vast majority of these SNVs, their functional consequences—pathogenic or benign—remains unknown. Experimental validation typically requires substantial time and resources, making it unsuitable for large-scale implementation. This widening gap underscores the pressing need to develop computational methods that are both efficient and accurate.

To address this challenge, numerous computational methods have been developed. These approaches can be broadly classified into three categories. The first category consists of methods based on evolutionary conservation and statistical analysis, such as SIFT (Ng and Henikoff 2003) and FATHMM (Shihab *et al.* 2014). These tools evaluate the impact of an amino acid substitution based on multiple sequence alignments. The second category encompasses a wide range of classical machine learning models, including CADD (Rentzsch *et al.* 2019), DANN (Quang *et al.* 2015), fathmm-MKL (Shihab *et al.* 2015), MetaSVM (Dong *et al.* 2015), and usDSM (Tang *et al.* 2021). These methods excel at integrating diverse, precomputed features such as allele frequencies and conservation scores to train predictive classifiers. More recently, deep learning-based methods have emerged as a third category, with tools like MetaRNN (Li *et al.* 2022), 3Cnet (Won *et al.* 2021), PrimateAI (Sundaram *et al.* 2018), MVP (Qi *et al.* 2021), and AlphaMissense (Cheng *et al.* 2023) leveraging neural networks to learn complex, nonlinear patterns directly from the data.

While the above approaches have advanced the field of variant pathogenicity prediction, they still face two primary limitations. (i) Biologically, the input data for pathogenicity prediction have the inherent gene-variant-feature association structure. That is, a single gene may harbor multiple variants, which are functionally linked due to their impact on the same gene, and each variant can be characterized by a vector of features assumed to reflect the likelihood of pathogenicity. Current methods fail to explicitly model the gene-variant-feature association, thus limiting their performance. (ii) Methodologically, the variant-pathogenicity association can be assumed to comprise a linear component and a nonlinear one. However, existing approaches generally rely on nonlinear models to characterize the relationship between genetic variants and pathogenicity, while overlooking its potential linear component. Because linear associations may be more appropriately captured by linear models, exclusively using nonlinear architectures can lead to suboptimal performance. Consequently, current methods may not effectively separate and model the linear effects that could be better represented through a linear framework.

To enhance variant pathogenicity prediction, we present SIMLINK, a method for the simultaneous modeling of linear and nonlinear components within a knowledge graph. The design of SIMLINK is guided by two principles. First, a knowledge graph provides an intuitive framework for representing the complex relationships between biological entities. We leverage this strength to integrate the inherent association structures among genes, variants, and features (Chen *et al.* 2023). Second, our approach explicitly isolates and models the linear component of the variant-pathogenicity association. This is achieved by introducing a linear model (Lin *et al.* 2024), in addition to a nonlinear one, to specifically capture the linear association from the data.

We applied SIMLINK to predict the pathogenicity of genetic variants. Our method begins with the construction of a variant-centered knowledge graph where each variant is characterized by a feature vector comprising allele frequencies, conservation scores, and pathogenicity predictions from existing methods, among others. Based on this graph and feature matrix, a hybrid model—consisting of a linear component and a graph neural network—is utilized to learn both the linear and nonlinear components of the variant-pathogenicity association. We trained SIMLINK using ClinVar variants (Landrum *et al.* 2018). We benchmarked SIMLINK against seven state-of-the-art (SOTA) methods across eight independent datasets, including seven missense variant datasets and one synonymous variant dataset. The results demonstrate that SIMLINK achieves superior prediction performance compared to SOTA methods such as CADD and MVP. To further evaluate its clinical potential, we assessed SIMLINK’s capacity to classify variants related to autism spectrum disorder (ASD). Functional characterization and supporting evidence from previous studies suggested that the variants prioritized by SIMLINK are functionally implicated in ASD. Collectively, these results demonstrate the potential of SIMLINK as a valuable tool for clinical variant prioritization.

## 2 Materials and methods

### 2.1 The SIMLINK method

**Figure 1:**
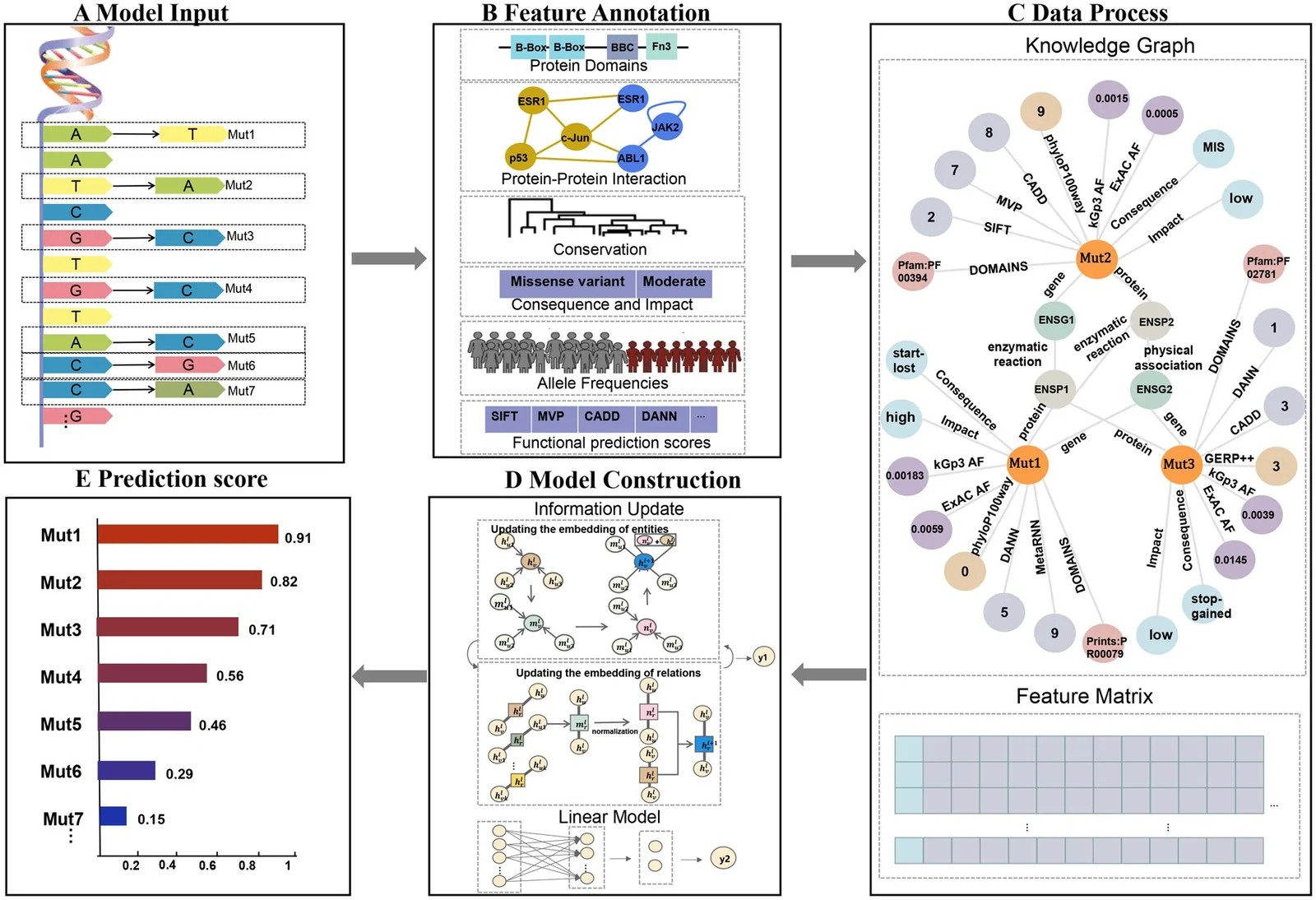
Overview of SIMLINK. (A) Input. Basic variant information, including chromosome, genomic position, reference allele, and alternate allele. (B) Annotation. Variants are annotated using six categories of biological features, including protein domains, protein–protein interactions, conservation scores, functional prediction scores, allele frequencies, and variant consequence annotations. (C) Construction of knowledge graph and feature matrices. The annotated information is transformed into a variant-centered knowledge graph, where variants are connected to genes and heterogeneous biological feature nodes. Corresponding feature matrices are constructed to represent biological attributes associated with each entity. (D) Model learning. The graph convolutional network updates entity representations by aggregating information from neighboring entities and their relationships within the knowledge graph, thereby capturing complex nonlinear interactions. In parallel, a linear model learns direct contributions from biological features. (E) Output. The outputs of the graph network and linear model are integrated through weighted fusion, and the resulting prediction score is used to estimate the pathogenicity of the variant.

SIMLINK predicts variant pathogenicity by leveraging a knowledge graph with a hybrid model that combines a graph convolutional network (GCN) and a linear model. This framework explicitly models the complex associations among genes, variants, and features by constructing a variant-centered knowledge graph, and innovatively integrates a linear model with a GCN to simultaneously analyze the linear and nonlinear components of pathogenicity, respectively. As shown in Fig. 1, its workflow comprises the following steps: collecting data on variants and molecular interactions, constructing the knowledge graph and feature matrices, training the prediction model by simultaneously learning a linear model and a GCN, and generating pathogenicity scores.

Specifically, six categories of biological annotations, including protein domains, protein-protein interactions, conservation scores, functional prediction scores, allele frequencies, and variant consequence annotations, are incorporated into the knowledge graph. Variants serve as central entities linking genes and heterogeneous biological feature nodes, enabling the model to capture both direct biological associations and higher-order relationships. During model training, the GCN learns complex nonlinear patterns among entities and relations, while the linear model captures linear patterns. The outputs of the two components are then integrated through weighted fusion to generate the final pathogenicity prediction score.

#### 2.1.1 Construction of knowledge graph

We construct a knowledge graph using data from multiple databases to capture the complex associations among variants, genes, proteins, and biological processes. The knowledge graph is built through a two-stage process. First, we extracted multiple types of information from the ClinVar (Landrum *et al.* 2018), Reactome (Fabregat *et al.* 2018), and dbNSFP (Liu *et al.* 2020) databases. Second, these data are transformed into triplets of the form (head entity, relation, tail entity), which define the graph’s relations by capturing entity interactions and associations. For example, (19_45411941_T_C, gene, APOE) indicates that the mutation from T to C at the 45 411 941 position on chromosome 19 is located within the gene APOE. In this graph, we consider the following biological entities and relations for constructing nodes and edges (see detailed processing and filtering criteria in Text 1, available as supplementary data at *Bioinformatics* online).

The nodes in the triplet cover various types of biological entities and annotation information related to variants, primarily including: (i) variant consequences and impact levels, (ii) genes, proteins, and transcripts, (iii) protein domains, (iv) genotypes, (v) amino acid types, (vi) conservation scores, and (vii) functional prediction scores.

The edges in the triplets represent the relations between their connecting nodes, which mainly include: (i) variant-gene relations, (ii) variant–pathogenicity score relations, (iii) variant–allele frequency relations, and (iv) protein–protein interaction relations.

The resulting knowledge graph contains 152 978 head entities, 73 661 tail entities, and 8 808 580 triplets.

#### 2.1.2 Model architecture

SIMLINK is an approach that integrates both linear and nonlinear models of knowledge graphs for predicting variant pathogenicity.

First, SIMLINK employs a graph neural network to capture nonlinear associations between genetic variants and pathogenicity from the knowledge graph. In this module, the initial representations of entities and relations are randomly sampled from a normal distribution. The feature for entities and relationships is initialized as a 128-dimensional vector following the convention (Kipf and Welling 2017).

In the knowledge graph, a GCN is used to update the embedding of entities and relations by gathering data from their neighbors. For each triplet, (u,r,v), where *u, r, v* denote head entity, relation, and tail entity respectively, let $h_{u}^{l},h_{r}^{l},h_{v}^{l}$ represent the embedding of entity *u*, relation *r*, and entity *v* at layer *l* respectively. Since triplets observed in the knowledge graph tend to have higher scores than those that have not been observed, we introduce a scoring function $f(h_{u}^{l},h_{r}^{l},h_{v}^{l})\to R$ to measure the plausibility of entity-relation-entity triplets. Most knowledge graph embedding techniques can be used to define *f*, such as TransE, DistMult, TransH, TransD, RotatE, and QuatE (Ji *et al.* 2022). We tested six of them in our model. Results from 10-fold cross-validation showed that QuatE performed the best. Therefore, we choose QuatE for this work.

The embedding of nodes is updated as follows. Considering that triplets possess directionality, we divide the neighbors of entity *v* into two groups: incoming links and outgoing links, enabling the GCN model to capture the relational directionality in the knowledge graph. The aggregated representation of *v’*s neighborhoods, $m_{v}^{l+1}$, is calculated as:


(1) $$\begin{array}{c} m_{v}^{l+1}=\sum_{\left(u,r)\in N_{in}(v\right)}W_{r}^{l}\frac{\partial f_{in}(h_{u}^{l},h_{r}^{l},h_{v}^{l})}{\partial h_{v}^{l}}\\ +\sum_{\left(u,r)\in N_{\mathrm{out}}(v\right)}W_{r}^{l}\frac{\partial f_{\mathrm{out}}(h_{u}^{l},h_{r}^{l},h_{v}^{l})}{\partial h_{v}^{l}} \end{array},$$

where $N_{in}(v)=\left\{(u,r)\;|u\overset{r}{\to }v\right\}$ is the set of immediate entity-relation neighbors of entity *v* with an in-link, $N_{\mathrm{out}}(v)=\left\{(u,r)\;|u\overset{r}{\leftarrow }v\right\}$ is the set of immediate neighbors with an out-link from *v*, and $W_{r}^{l}$ denotes the relation-specific linear transformation matrix. The scoring function $f_{in}$ is for the in-link neighbors and $f_{\mathrm{out}}$ is for the out-link neighbors, respectively. Then a normalization operation is applied:


(2) $$n_{v}^{l+1}=\theta \frac{m_{v}^{l+1}}{\sum_{i\in N_{in}(v)}m_{i}^{l+1}+\sum_{j\in N_{\mathrm{out}}(v)}m_{j}^{l+1}},$$

where $n_{v}^{l+1}$ is the aggregated representation of *v’*s neighborhoods after normalization, and θ is a hyperparameter that controls the weight of aggregated neighbor information for entity update. The rule for updating the embedding of entity *v* is as follows:


(3) $$h_{v}^{l+1}=\sigma_{\mathrm{ent}}(W_{\mathrm{ent}}^{l}(n_{v}^{l+1}+h_{v}^{l})),$$

where $\sigma_{\mathrm{ent}}$ denotes the activation function for entity update. The convolutional process updates feature representations by aggregating and transforming local neighborhood information.

The embedding of relations is updated in a similar manner. The following rule is used to update the embedding of relation *r*:


(4) $$m_{r}^{l+1}=\sum_{\left(u,v)\in N(r\right)}\frac{\partial f_{r}(h_{u}^{l},h_{r}^{l},h_{v}^{l})}{\partial h_{r}^{l}},$$

(5) $$n_{r}^{l+1}=\gamma \frac{m_{r}^{l+1}}{\sum_{k\in N(r)}m_{k}^{l+1}},$$

(6) $$h_{r}^{l+1}=\sigma_{\mathrm{rel}}(W_{\mathrm{rel}}^{l}(n_{r}^{l+1}+h_{r}^{l})),$$

where $N(r)=\left\{(u,v)\;|u\overset{r}{\to }v\right\}$ means the set of immediate entity neighbors of relation r, where the left side of tuple is head entity and the right side is tail entity. γ is a hyperparameter that controls the weight of aggregated neighbor information for relation update. $\sigma_{\mathrm{rel}}$ denotes the activation function for relation update.

During end-to-end optimization, SIMLINK is trained exclusively using the binary cross-entropy loss for pathogenicity prediction. The scoring function is not associated with an additional knowledge graph embedding objective; instead, its gradient is used only to determine the direction of message propagation.

To compute the pathogenicity score $P_{v}$ of a variant denoted by *v*, a sigmoid function is used to generate the final output:


(7) $$P_{v}=\frac{1}{1+e^{-{\tilde{h}}_{v}^{L}}},$$

where ${\tilde{h}}_{v}^{L}$ is the entity embedding of the final layer and L is the number of layers.

Second, SIMLINK uses a linear model to characterize the linear effects underlying the relationship between genetic variants and pathogenicity. The model projects the input features onto a low-dimensional latent representation and generates an output score between 0 and 1, where a higher score denotes a higher probability of the variant being pathogenic. The linear module is expressed as follows:


(8) $$y_{2}=X\beta ,$$

where X is the feature matrix constructed from the numerical features used as input to the nonlinear module, after median imputation and normalization, β denotes the learnable linear coefficients.

Third, the final output *y* of the model combines the results from both the nonlinear module $y_{1}=P_{v}$ and the linear module $y_{2}$. It is defined by the following formula:


(9) $$y=\alpha y_{1}+(1-\alpha )y_{2},$$

where α∈[0,1] is a weighting parameter. A larger α value means a higher proportion of the nonlinear model in the final output. Experimental results demonstrate that this collaborative integration strategy of linear and nonlinear models effectively enhances predictive performance.

### 2.2 Datasets

In this work, nine datasets (see details in Table 1) are used for model training, validation, and testing. These datasets are briefly described below.

**Table 1:** The summary of the nine variant datasets.^a^

Dataset	Type	Pathogenic	Benign	Total	Usage
ClinVar	Missense	58 159	49 043	1 07 202	Training
HumVar	Missense	19 294	16 722	36 016	Validation
ClinVar2	Missense	6914	24 157	31 071	Testing
TP53	Missense	393	552	945	Testing
ExoVar	Missense	4777	3117	7894	Testing
VariBench	Missense	4146	5137	9283	Testing
PredictSNP	Missense	8981	5138	14 119	Testing
SwissVar	Missense	3922	6590	10 512	Testing
dbDSM	Synonymous	34	792	826	Testing

a To prevent information leakage, any variants shared between the training and test datasets were excluded from the test set.

Training and validation datasets: The ClinVar variant dataset (June 2022 release) is used for model training, the HumVar dataset for tuning the parameter α.

Testing datasets: To avoid information leakage, any variants shared between the training and test datasets were excluded from the test set. ClinVar2 (derived from the March 2023 ClinVar update) includes newly discovered clinical variants not covered by the training set (its release date postdates all functional prediction scoring methods). The TP53 test set focuses on key tumor-related genes, specifically evaluating the ability of different methods to predict pathogenicity of tumor-associated missense mutations (Fromentel and Soussi 1992). Meanwhile, ExoVar, PredictSNP, SwissVar, and VariBench are widely used benchmark datasets in missense mutation pathogenicity research; their differences in data sources, mutation type distributions, and pathogenicity annotation standards enable comprehensive reflection of predictive performance across diverse scenarios (Grimm *et al.* 2015). Additionally, the dbDSM test set, which is curated from a high-quality database, is used to evaluate the predictive performance on synonymous variants (Wen *et al.* 2016). The details for processing each dataset are provided in Text 2, available as supplementary data at *Bioinformatics* online.

## 3 Results and discussion

### 3.1 Comparison with state-of-the-art pathogenicity prediction methods

We constructed the SIMLINK model using the gold-standard ClinVar training dataset. To determine the optimal value for the parameter α, we evaluated a series of values: 0, 0.1, 0.3, 0.5, 0.7, 0.9, and 1. Our experiments on the HumVar validation dataset, which contains the largest number of variants among all test sets, showed that the best performance is achieved when α = 0.5 (Fig. 1, available as supplementary data at *Bioinformatics* online). This result for parameter α shows that when the model relies solely on the linear module (α=0) or solely on the nonlinear GCN module (α=1), the performance fails to reach its optimum. Both AUROC and AUPRC peak only when the two modules are balanced (α=0.5), highlighting the complementary contributions of the linear and graph modules. We further evaluated different α values across all benchmark datasets, and the corresponding results are summarized in Table 1, available as supplementary data at *Bioinformatics* online. Although the optimal α value varies across datasets, intermediate α values between 0 and 1 generally achieve better performance than either extreme setting, further supporting the effectiveness of integrating the graph-based and linear modules.

First, we compare SIMLINK with existing methods in predicting the pathogenicity of missense variants. The six test sets, namely ClinVar2, TP53, ExoVar, PredictSNP, VariBench, and SwissVar, are used. The benchmark methods comprise seven prediction methods, including Eigen, PrimateAI, MVP, MetaRNN, CADD, DANN, and fathmm-MKL. Pathogenicity scores predicted by these methods were extracted from the dbNSFP v4.3a database (Liu *et al.* 2020). Table 2 presents the area under the receiver operating characteristic curve (AUROC) and the area under the precision-recall curve (AUPRC) for comparison. The standard error was estimated based on ten times resampling of test sets. The results demonstrate that SIMLINK consistently achieves superior performance, obtaining the highest AUROC scores on five out of the six datasets. Notably, SIMLINK reaches exceptional scores of 0.983 on ClinVar2, 0.963 on PredictSNP, and 0.995 on the ExoVar dataset. While MVP records the top score on the VariBench dataset, SIMLINK consistently outperforms other leading methods like MetaRNN and MVP across the majority of the benchmarks, establishing its robustness and superior predictive power for missense variant pathogenicity. Based on our results and the dataset summary statistics in Table 1, SIMLINK shows clearer improvements on ClinVar2 and SwissVar. Both datasets contain relatively large numbers of variants. In contrast, SIMLINK does not show an improvement on the smaller TP53 dataset. This observation suggests that sample size might be a potential factor affecting prediction performance; a possible reason is that larger datasets contain more gene-variant-feature associations, allowing SIMLINK to make better use of the knowledge graph for prediction.

**Table 2:** Performance comparison of different methods for missense variant pathogenicity prediction using AUROC and AUPRC^a^.

Methods	ClinVar2	TP53	PredictSNP	VariBench	ExoVar	SwissVar
**AUROC**						
DANN	0.8255±0.0006	0.7288±0.0042	0.7218±0.0013	0.6316±0.0022	0.7798±0.0016	0.6889±0.0011
fathmm-MKL	0.8369±0.0006	0.7851±0.0028	0.7447±0.0010	0.6498±0.0015	0.8162±0.0016	0.7197±0.0018
PrimateAI	0.8530±0.0009	0.8220±0.0036	0.7502±0.0014	0.6717±0.0015	0.8389±0.0016	0.7341±0.0015
MVP	0.9423±0.0004	0.8463±0.0026	0.9532±0.0005	**0.9257** ± **0.0010**	0.9647±0.0005	0.7286±0.0017
MetaRNN	0.9723±0.0002	**0.8867** ± **0.0027**	0.9462±0.0005	0.8674±0.0014	0.9888±0.0004	0.8395±0.0014
CADD	0.9086±0.0008	0.8196±0.0041	0.7963±0.0011	0.6677±0.0017	0.8726±0.0016	0.7431±0.0013
Eigen	0.9201±0.0006	0.8298±0.0032	0.8102±0.0009	0.6536±0.0020	0.8692±0.0016	0.7421±0.0012
SIMLINK	**0.9825** ± **0.0002**	0.8739±0.0031	**0.9630** ± **0.0004**	0.8877±0.0013	**0.9953** ± **0.0002**	**0.8504** ± **0.0009**
**AUPRC**						
DANN	0.4662±0.0020	0.5866±0.0070	0.7767±0.0015	0.5512±0.0031	0.8071±0.0025	0.5254±0.0028
fathmm-MKL	0.5010±0.0017	0.6936±0.0068	0.7974±0.0012	0.5813±0.0024	0.8408±0.0013	0.5583±0.0031
PrimateAI	0.6002±0.0020	0.7673±0.0073	0.8218±0.0015	0.5781±0.0027	0.8750±0.0017	0.6306±0.0022
MVP	0.8467±0.0013	0.8231±0.0042	0.9757±0.0003	**0.9093** ± **0.0017**	0.9763±0.0004	0.6650±0.0027
MetaRNN	0.9198±0.0007	**0.8608** ± **0.0030**	0.9705±0.0004	0.8455±0.0017	0.9943±0.0002	0.7548±0.0023
CADD	0.6950±0.0030	0.6989±0.0062	0.8387±0.0018	0.5976±0.0032	0.9017±0.0013	0.6068±0.0024
Eigen	0.7570±0.0023	0.7421±0.0072	0.8559±0.0012	0.5914±0.0038	0.8931±0.0014	0.6069±0.0026
SIMLINK	**0.9519** ± **0.0007**	0.8423±0.0054	**0.9793** ± **0.0003**	0.8702±0.0016	**0.9973** ± **0.0001**	**0.7973** ± **0.0019**

^a^The best result for each dataset under each evaluation metric is highlighted in bold.

**Figure 2:**
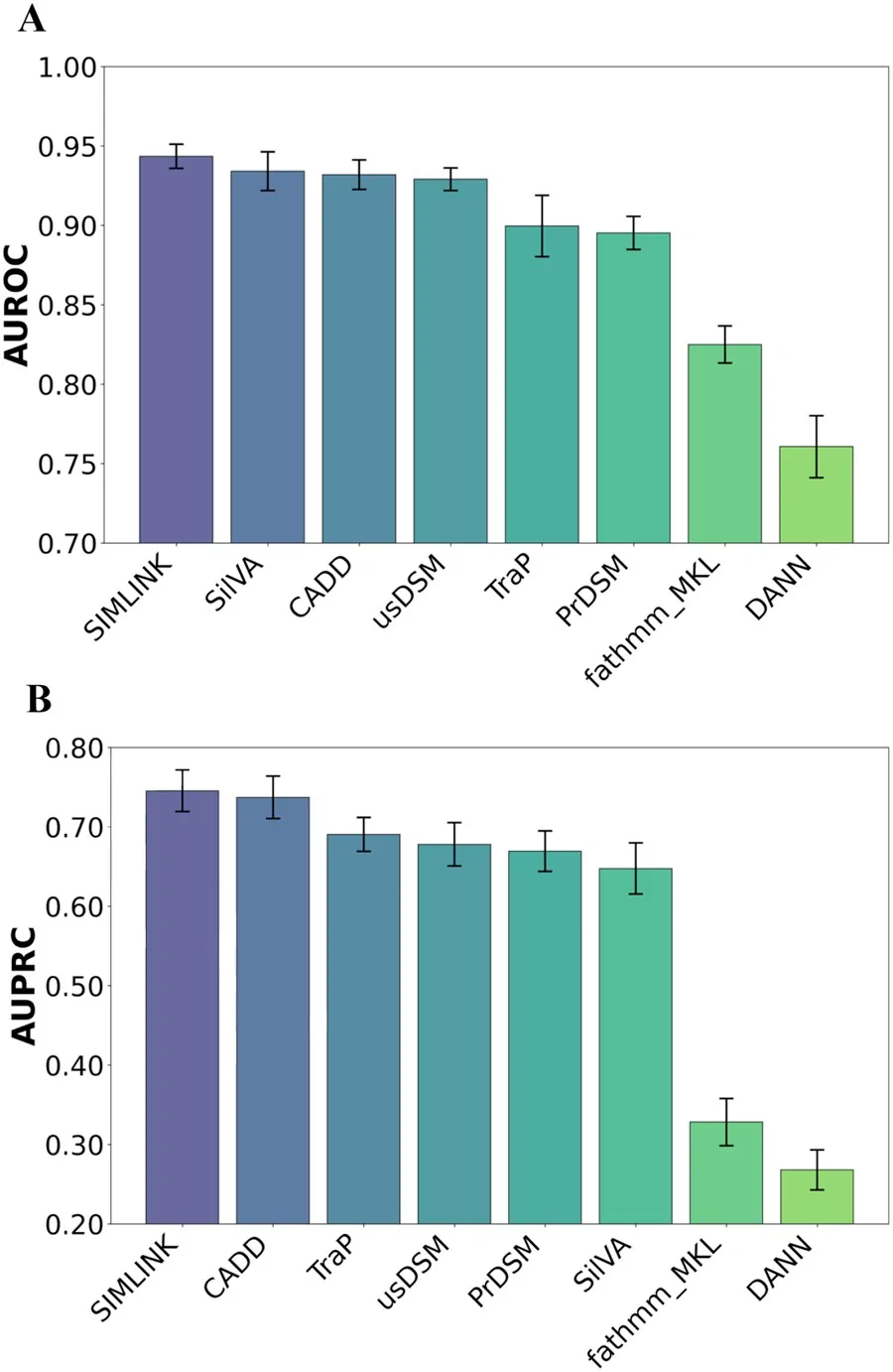
Performance comparison of synonymous variant prediction. (A) AUROC results. (B) AUPRC results.

Second, we compare SIMLINK with existing methods in predicting the pathogenicity of synonymous variants. The dbDSM is used to evaluate the performance of SIMLINK against four dedicated methods, SilVA (Buske *et al.* 2013), TraP (Gelfman *et al.* 2017), PrDSM (Cheng *et al.* 2020), and usDSM, and three general-purpose methods, CADD, DANN, and fathmm-MKL. Among these, the pathogenicity scores for SilVA, TraP, PrDSM, and fathmm-MKL are obtained from the PrDSM website, while the scores for usDSM, CADD, and DANN are obtained from their respective official online servers. Figure 2 presents the comparative performance of the evaluated methods, with subfigure A showing the AUROC results and subfigure B showing the AUPRC results. SIMLINK achieves the highest predictive accuracy with a leading AUROC of 0.9154. Following closely are CADD and usDSM, which also exhibit strong predictive power with AUROC of 0.8998 and 0.8957, respectively. Methods such as PrDSM, TraP, and SilVA deliver moderate performance, while fathmm-MKL and DANN show the lowest efficacy in this comparison, underscoring the significant variability in the predictive capabilities of current tools for this specific class of variants.

Third, we compare SIMLINK with AlphaMissense. It is a leading deep learning model that leverages structural context from AlphaFold and evolutionary constraints to predict the pathogenicity of missense variants. It combines unsupervised protein language modeling with fine-tuning on population frequency data to achieve high predictive accuracy. In this study, we retrieved pathogenicity scores from the AlphaMissense database (https://doi.org/10.5281/zenodo.8208688). We extract the intersection of variants predicted by both SIMLINK and AlphaMissense, and rename the resulting datasets SwissVar*, VariBench*, PredictSNP*, ClinVar2*, ExoVar*, and TP53*. For genomic positions containing multiple prediction entries, we selected the maximum score as the final prediction for that site.

To further evaluate the robustness and stability of the comparison results, we performed five repeated random resampling experiments for each dataset and reported the mean AUROC and AUPRC together with their corresponding standard errors.

Detailed experimental results, as shown in Table 3, demonstrate that SIMLINK achieves advantages across both AUROC and AUPRC metrics in five out of the six independent test datasets. The standard errors are consistently small across most datasets, indicating that SIMLINK maintains stable predictive performance under different data partitions and repeated evaluations.

**Table 3:** Performance comparison between SIMLINK and AlphaMissense across five repeated evaluations.^a^

	AUROC		AUPRC	
Dataset	SIMLINK	AlphaMissense	SIMLINK	AlphaMissense
SwissVar	**0.8512** ± **0.0024**	0.7606±0.0027	**0.8007** ± **0.0033**	0.6861±0.0029
VariBench	**0.8846** ± **0.0017**	0.7026±0.0039	**0.8683** ± **0.0018**	0.6680±0.0016
PredictSNP	**0.9630** ± **0.0008**	0.8111±0.0028	**0.9796** ± **0.0006**	0.8685±0.0024
ClinVar2	**0.9825** ± **0.0010**	0.9523±0.0020	**0.9524** ± **0.0020**	0.8687±0.0043
ExoVar	**0.9953** ± **0.0003**	0.9117±0.0045	**0.9974** ± **0.0002**	0.9472±0.0028
TP53	0.8892±0.0201	**0.9195** ± **0.0138**	0.8520±0.0282	**0.9027** ± **0.0119**

a Datasets marked with a star * consist of the intersection of variants predicted by both SIMLINK and AlphaMissense. The best results are highlighted in bold. Results are reported as mean ± standard error.

### 3.2 Effect of allele frequency on prediction performance

**Figure 3:**
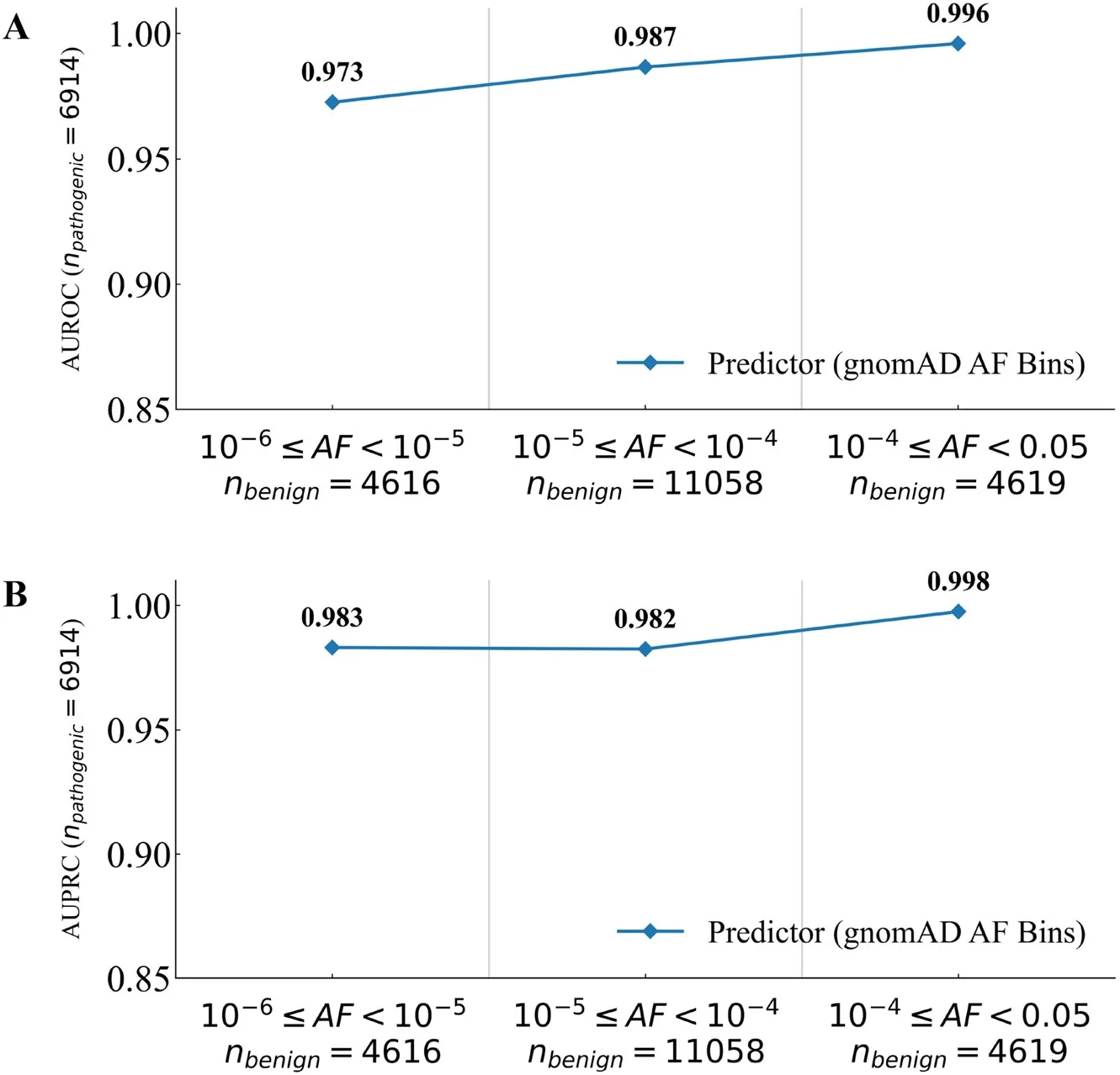
Performance across different allele frequency bins. (A) AUROC results. (B) AUPRC results.

We evaluated the impact of allele frequencies following the method proposed in (Rastogi *et al.* 2025). First, we identify a set of 6914 pathogenic variants from the ClinVar2 dataset, and use this set for evaluation. Second, we use the gnomAD reference database to extract allele frequencies. gnomAD provides the frequency of variants in the general population. Third, we group the benign variants into three bins by their allele frequencies from gnomAD following the approach in (Rastogi *et al.* 2025). We measure the performance for each of these three combined groups. SIMLINK maintains high performance across all frequency groups. Both AUROC and AUPRC scores stay above 0.97 in every group (Fig. 3). The performance increases as the benign variant frequency rises. These results demonstrate the high predictive power of SIMLINK.

### 3.3 Feature importance

**Figure 4:**
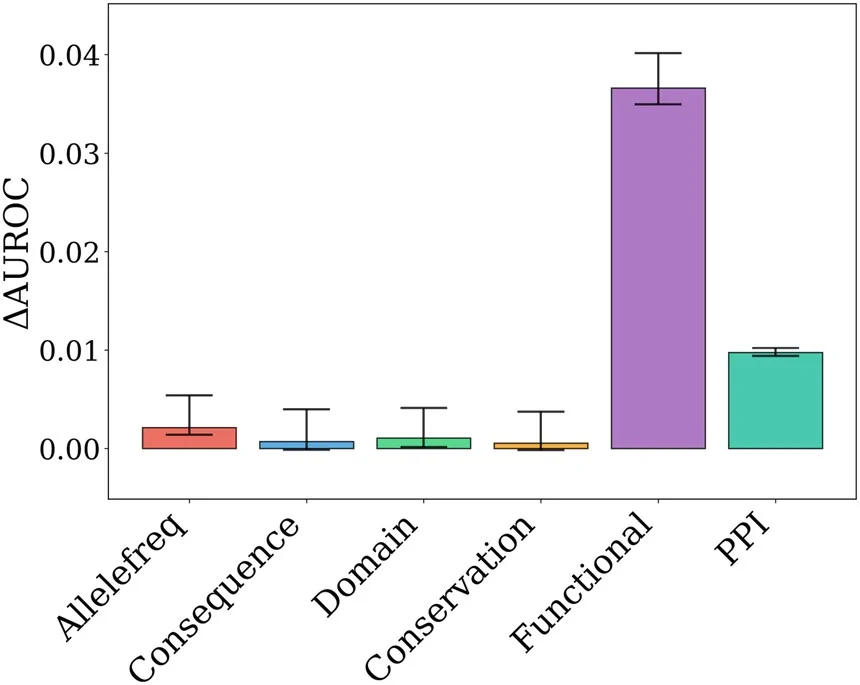
Importance of the six feature groups in terms of ΔAUROC.

We perform ablation experiments to evaluate the importance of features. We classify the features into six groups based on their functional categories. These groups include Allele Frequency, Consequence, Domain, Conservation, Functional, and PPI. Specifically, the Allele Frequency group consists of three population frequency features: the 1000 Genomes Project Phase 3 allele frequency, the ExAC allele frequency, and the gnomAD exomes allele frequency. The Consequence group includes variant consequence annotations, capturing the predicted functional impact of each variant on gene transcripts, such as missense, nonsense. The Domain group includes protein domain annotations from databases such as Pfam, PANTHER, Gene3D, PROSITE, SMART, and Superfamily. The Conservation group comprises two evolutionary conservation scores derived from 100-vertebrate species comparisons. The Functional group includes a set of pathogenicity prediction scores from multiple algorithms, such as CADD, DANN, Eigen, FATHMM, MetaRNN, PrimateAI, and SIFT. The PPI group includes protein-protein interaction network. Due to the high correlation among features within the same group, we iteratively remove all features within a single group and evaluate the performance on the ClinVar2 data. We choose ClinVar2 because these variants are of the highest quality. By calculating the AUROC difference between the full model and with a feature group removed, we quantify the contribution of each feature group based on the magnitude of performance degradation, thereby identifying the most influential features (Fig. 4). The results clearly indicate that the functional feature group is the most critical, contributing to a substantial increase in model accuracy. The PPI features also provide a significant positive impact. In contrast, the remaining feature groups demonstrate a negligible effect.

### 3.4 Consistency of pathogenicity prediction methods

**Figure 5:**
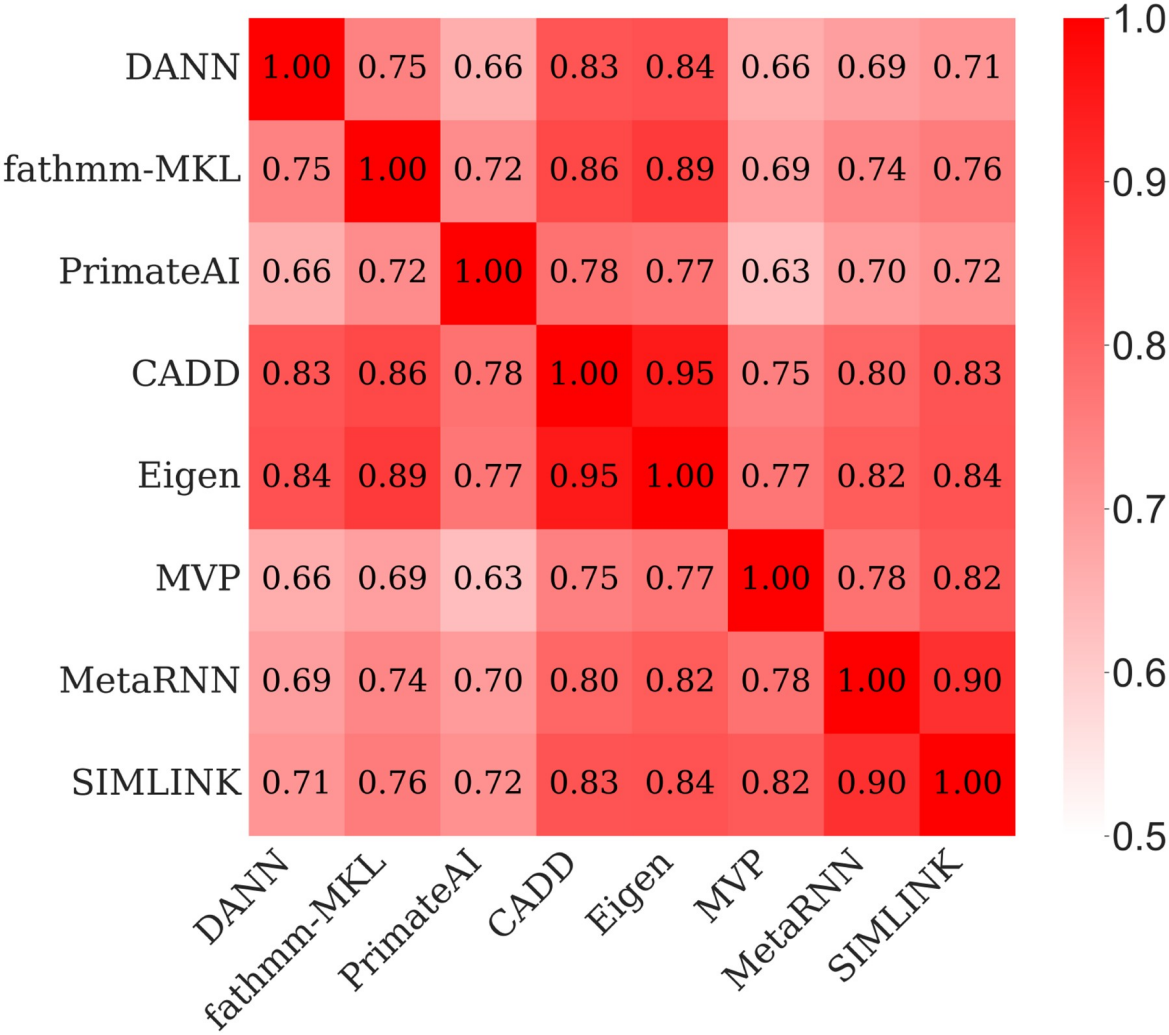
The consistency between methods based on their prediction scores on the ClinVar2 test set.

We evaluate the consistency between SIMLINK and existing methods. We calculate the Spearman correlation coefficients of each method’s prediction scores on the ClinVar2 dataset and visualize the results as a heatmap (Fig. 5). The analysis reveals strong positive correlations among most prediction methods, indicating good consistency and reliability among different computational methods. Specifically, SIMLINK and MetaRNN demonstrate the highest correlation (ρ = 0.90). This high correlation suggests that the two methods may share significant similarities in the way of using training data. Meanwhile, the association between SIMLINK and Eigen is also relatively strong (ρ = 0.84). Evaluation of six additional datasets including HumVar, TP53, ExoVar, VariBench, PredictSNP, and SwissVar shows that these correlation coefficients are stable across different data sources (Fig. 2, available as supplementary data at *Bioinformatics* online). These substantial positive correlations indicate that although these methods employ different computational strategies, they share considerable consensus in predicting variant pathogenicity.

### 3.5 Prioritizing *de novo* missense variants in autism spectrum disorder

We evaluate the practical utility of variant pathogenicity prediction approaches using a case study of autism spectrum disorder (ASD) variants. We use the dataset comprising 2396 *de novo* missense variants (Iossifov *et al.* 2014) from 3953 ASD cases and 975 *de novo* missense variants from 1911 controls (unaffected siblings) in the Simons Simplex Collection (De Rubeis *et al.* 2014).

**Figure 6:**
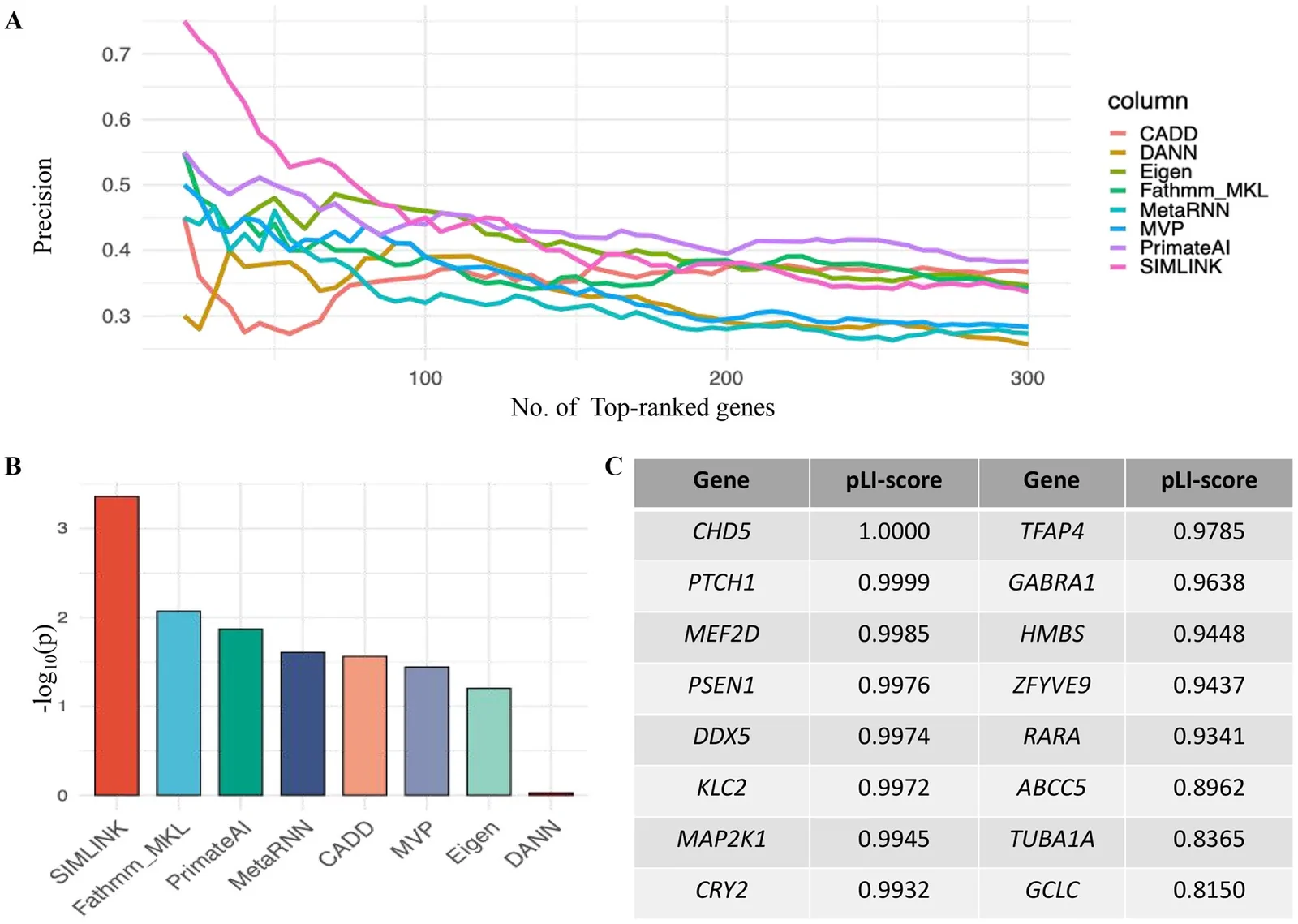
Performance of SIMLINK in prioritizing ASD-Associated genetic variants. (A) Comparison of the overlap of top predicted genes with known ASD genes. (B) Comparison of the performance in distinguishing between high-confidence and low-confidence (i.e. with suggestive confidence) ASD genes. The higher $-{\;\text{log}\;}_{10}(p)$ is, the more significant. (C) The top ranked novel (not in SFARI) ASD candidate genes prioritized by SIMLINK have high pLI score.

First, we evaluate the ability of various computational methods to differentiate between high-confidence (score = 1) and low-confidence (score = 3, suggestive evidence) ASD genes in the SFARI database. Figure 6 shows the statistical significance (−log_10_(*P* value)) of this comparison, with the *P* value calculated using the Mann Whitney U test. SIMLINK achieves the highest significance level (P<.01), outperforming seven other state-of-the-art methods. This result highlights SIMLINK’s enhanced capability to accurately rank established ASD-risk genes based on the strength of existing evidence. The results of various computational methods used to distinguish ASD genes in the SFARI database for all data are shown in Fig. 3, available as supplementary data at *Bioinformatics* online.

Second, the top-ranked genes by SIMLINK are biologically associated with ASD, both within and beyond the SFARI database. For example, CHD8 (rank score = 1.000) is a gene associated with ASD with high confidence (Category 1) in SFARI (Sugathan *et al.* 2014). Heterozygous loss-of-function or truncating mutations in CHD8 are strongly linked to characteristic phenotypes including macrocephaly, developmental delay, and ASD. Consistent with this, animal and cellular models demonstrate that CHD8 haploinsufficiency disrupts developmental transcriptional networks. Another example is DENR. Mutations in this gene selectively impair the local translation of neuron-specific transcripts (Haas *et al.* 2016), thereby leading to defects in neuronal migration and development. This mechanism may underlie the neurodevelopmental abnormalities observed in reported ASD cases.

Third, SIMLINK prioritizes novel ASD candidate genes with supportive evidence. After removing all genes in SFARI, we examined the probability of Loss-of-function Intolerant (pLI) scores of the top-ranked 30 genes. The pLI score is an independent measure of a gene’s intolerance to functional mutations, where a score close to 1.0 indicates a high likelihood of the gene to be pathogenic. Of these, 16 genes exhibit high pLI scores higher than 0.80 (ranging from 0.815 to 1.000), including two genes, CHD5 and COL5A2, with the maximum pLI score of 1.0. We assessed the statistical significance of this enrichment using a permutation test. Specifically, 30 genes were randomly sampled 10 000 times from 2964 non-SFARI genes with available pLI scores. Only 31 random samples contained at least as many genes with pLI>0.8 as those prioritized by SIMLINK, yielding a significant empirical *P* value of.0031. Notably, CHD5 encodes a neuron-specific chromatin remodeler and has been implicated in autism spectrum disorder through its role in regulating neuronal migration and differentiation (Goodman and Bonni 2019). Heterozygous CHD5 variants have been identified in individuals with a neurodevelopmental syndrome characterized by intellectual disability, speech delay, epilepsy, and behavioral disturbances including autism spectrum disorder (Parenti *et al.* 2021). COL5A2 has been identified as a moderate-risk gene for autism spectrum disorder, with a rare missense variant reported in a large-scale sequencing study of 42 607 ASD cases (Zhou *et al.* 2022). Additionally, a patient with a *de novo* deletion encompassing COL5A2 presented with autism and intellectual disability, further supporting its potential role in neurodevelopmental disorders (Kempers *et al.* 2022). This independent biological evidence supports these novel candidates and underscores the effectiveness of SIMLINK in identifying potentially pathogenic genes for ASD.

**Figure 7:**
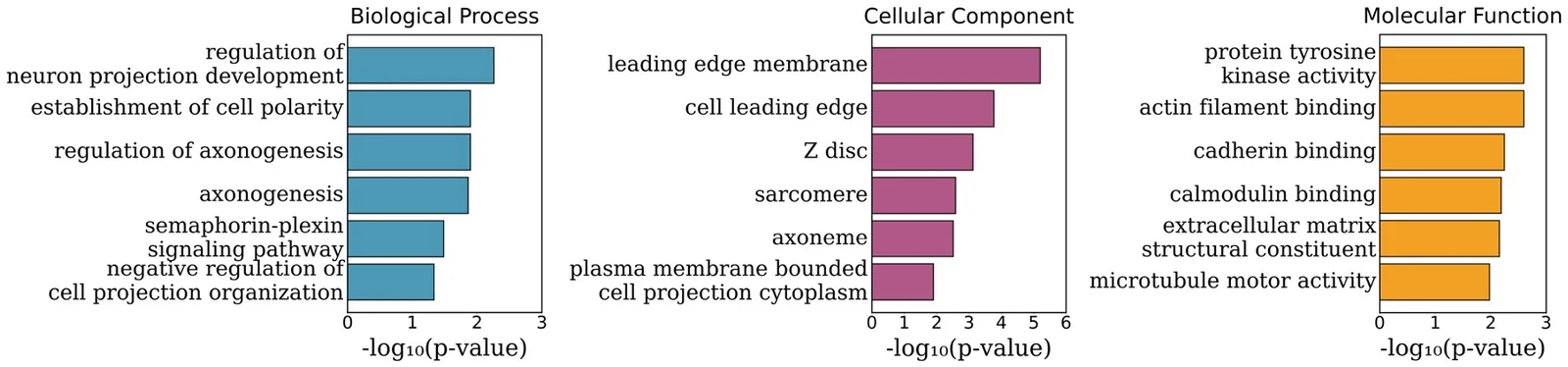
Enriched GO terms of prioritized ASD genes.

Fourth, functional enrichment was performed to test the biological relevance of the identified pathogenic variants. We first removed the genes in Categories 1, 2, and S in SFARI which are known to be associated with ASD. We then performed Gene Ontology (GO) enrichment analysis on the top-ranked genes (Fig. 7). We find that these genes are significantly enriched in ASD-related functions. For biological processes, the significant enrichment in terms such as axonogenesis (Xu *et al.* 2018), regulation of axonogenesis (Garcia-Forn *et al.* 2020), and regulation of neuron projection development (Willsey *et al.* 2013) indicates that these genes may participate in the aberrant neuronal connectivity observed in ASD. For cellular components, these genes are enriched in terms including cell leading edge (Pinto *et al.* 2010), leading edge membrane (Lilja *et al.* 2017), and plasma membrane bounded cell projection cytoplasm (Lin *et al.* 2016). The enriched molecular functions include cadherin binding, protein tyrosine kinase activity (Li *et al.* 2025), and calmodulin binding (Zhang *et al.* 2025), which are reportedly associated with ASD. Collectively, these findings suggest that the prioritized pathogenic variants under study are involved in fundamental processes of neuronal development, cell motility, and cytoskeletal organization.

## 4 Conclusion

While current methods for predicting variant pathogenicity have advanced the field, they still face limitations. They often fail to explicitly model the inherent gene-variant-feature association structure, and their reliance on predominantly nonlinear models can be suboptimal for describing the linear components of the variant-pathogenicity relationship. To address these gaps, we developed SIMLINK, a novel approach that uses a knowledge graph to represent these complex biological relationships and a hybrid model to simultaneously learn both linear and nonlinear associations. We compare SIMLINK to existing approaches including CADD, MVP, and MetaRNN on a total of seven test sets. The results indicate that our method performs better in most cases. In addition, we also performed a case study on ASD variants. We find that SIMLINK is better in distinguishing between high-confidence and low-confidence genes associated with ASD.

Despite its promising results, SIMLINK has several limitations that would be considered in our future work. First, model performance depends on the quality and coverage of the biological data used to construct the knowledge graph. Incorporating additional complementary biological data may provide a more comprehensive representation of variant effects. Meanwhile, increasing graph size might bring about potential computational and scalability concerns on building and managing more elaborate graphs. To address this concern, we plan to consider a two-fold strategy: (i) Graph Construction: we plan to enrich the graph by integrating complementary or orthogonal data types, such as expression quantitative trait loci (eQTLs) and alternative splicing-level data, which provide additional functional information without heavily increasing graph size, (ii) Graph Management: To ensure scalability, we will employ sampling-based graph convolutional network methods to process large-scale graphs efficiently. Additionally, we tested the computational cost of our approach. Training on the ClinVar dataset takes approximately 2.5 h on our server (Intel Xeon Gold 6330, 112 cores, 512 GB RAM), demonstrating that our approach is computationally efficient. Second, the neural network component presents a challenge to interpretability. Future studies will therefore investigate visualization and attribution methods to clarify how individual variants and biological relationships contribute to the predictions. We also plan to extend SIMLINK to additional variant types, including insertions, deletions, and structural variants, and to evaluate its generalizability across a broader range of diseases.

## Supplementary Material

btag601_Supplementary_Data

## Data Availability

The source code of SIMLINK is freely available at GitHub (https://github.com/Chen-LuWang/SIMLINK). The software version (v1.0.0), benchmark resources, and related data used in this study have been archived in Zenodo (https://doi.org/10.5281/zenodo.21802211).
