# Supplementary material for: SIMLINK enables accurate variant pathogenicity prediction through modeling the gene-variant-feature association structure
Source: Bioinformatics. 2026 Aug 10;42(8):btag601. doi: 10.1093/bioinformatics/btag601 (PMC13505626; doi:10.1093/bioinformatics/btag601)
Supplement: Supplementary file 1 [file btag601_supplementary_data.pdf]

Appendix：

**Material ID**	**Brief Summary**
**Text S1**	Entity and relation selection criteria.
**Text S2**	Detailed description of data processing.
**Figure S1**	Parameter optimization for SIMLINK model.
**Figure S2**	Consistency analysis of SIMLINK and other methods across six datasets.
**Figure S3**	The precision of the top-ranked genes for each method.
**Table S1**	Performance evaluation under different α values across independent benchmark datasets.

Text S1: Entity and relation selection criteria.

The entities of the knowledge graph include various biological entities and annotation information related to variants, mainly including the following: (1) Consequence and Impact. The variant consequences include `missense-variant', `start-lost', `stop-gained', and `synonymous-variant'. Impact indicates the severity of consequences including `low', `moderate', and `high'. (2) Allele frequencies. Allele frequencies were acquired from several databases, such as gnomAD[1] and ExAC[2]. (3) Protein domains. We collected the names of overlapping protein domains from Pfam[3] and InterPro[4] databases. When a protein existed in a protein database but the variant position did not fall into a known protein domain, it was considered as `outside' of the domain, and when a protein was absent from both Pfam and InterPro databases, it was considered as an `unknown' domain[5]. (4) Genotype. We collected the phenotype or the trait of the variant-related gene from DisGeNET database[6]. (5) Amino acids. The reference and alternative amino acid types were extracted by using VEP. (6) Conservation scores. GERP++[7], PhastCons 100way vertebrate[8], and PhyloP 100way vertebrate[9] were collected from dbNSFP database. (7) Functional prediction scores. Several functional prediction scores for missense variants, including CADD[10], DANN[11], fathmm-MKL[12], MetaSVM[13], MVP[14], Eigen[15], MetaRNN[16], Polyphen2-HDIV[17], Polyphen2-HVAR[17], MutationTaster[18], ClinPred[19], BayesDel[20], VEST4[21], DEOGEN2[22], PROVEAN[23] are collected from dbNSFP.4 functional prediction scores for synonymous variants, including SilVA[24], TraP[25], PrDSM[26], fathmm-MKL were collected from the website of PrDSM (http://bioinfo.ahu.edu.cn:8080/PrDSM).

Knowledge graphs are multi-relational graphs whose nodes represent entities and edges represent different relations between entities. The relationships in the knowledge graph are constructed by converting the annotation information of variants into triplets of the form (head entity, relationship, tail entity), reflecting interactions and associations between different entities, mainly including the following:

(1) The relationship between variants and genes. Variants and their annotated gene identifiers are used as the head entity and the tail entity respectively, and 'gene' is used as the relationship. For example, (19_45411941_T_C, gene, APOE) indicates that the mutation from T to C at the 45411941th position on chromosome 19 is located within the gene APOE.

(2) The relationship between variants and pathogenicity scores. We normalized pathogenicity scores using MinMaxScaler, defined as follows:

$$x' = \frac{x - x_{min}}{x_{max} - x_{min}}$$

where x' and x correspond to the score values after normalization and original scores values, where x_{max} and x_{min} mean the maximum and minimum value of the score values. MinmaxScaler can scale the interval, and convert data to [0, 1] based on the maximum and minimum values. We then convert the continuous values to discrete

values by multiplying the normalized value by 10 and keeping only the integer part. Finally, we store the data as triplets. For example, (1_949511_G _T, SIFT_converted _rankscore, 9) is a pathogenicity score triplet. In the triplet, `1 _949511 _G _T' is the head entity which is a key identifier of the variant composed of chromosome, position, reference nucleotide allele, and alternative nucleotide allele. `SIFT _converted _rankscore' and `9` correspond to the relation and the tail entity, which means that the variant is predicted by SIFT to be more likely to be damaging than 90 % of all possible variants in dbNSFP.

(3) The relationship between variants and allele frequency. We define variants with AF greater than 1% as common, variants with AF between 0 and 1% as rare, and variants with AF = 0 as de novo. Then, we take the variant and the type as the head and tail entity, respectively, and the source of allele frequency as the relationship. For example, (1 _865694_C_T, ExAC_AF, common) is an allele frequency triplet, which means that the variant is a common variant in ExAC database.

(4) The interactions between proteins. We downloaded protein-protein interaction data from Reactome (https://reactome.org/download/current/interactors). We treat two interacting proteins as head entities and tail entities, respectively, and the interactions between them as relationships. As for other variant annotation information, we store all the data as triplets. Finally, a knowledge graph consisting of 152,978 head entities, 73,661 tail entities, and 8,808,580 triplets was constructed.

Text S2：Detailed description of data processing.

The training data was sourced from the ClinVar database released in June 2022[27]. We download the June 2022 release of the ClinVar database under the GRCh37 genome build from https://ftp.ncbi.nlm.nih.gov/pub/clinvar/ to construct the training set. To obtain reliable training data with molecular consequences, we filter for variant records classified as 'single nucleotide variant' and having a reliable review status, such as 'reviewed by expert panel', `criteria provided' and `multiple submitters with no conflicts'. Variants labeled as `Pathogenic' or `Likely pathogenic' serve as the positive training set, while those labeled as `Benign' or `Likely benign' constitute the negative training set. Variants of uncertain clinical significance are excluded. After this rigorous filtering process, a total of 107,202 variants with clear pathogenicity labels are included for model training.

To conduct a comprehensive and unbiased performance evaluation, we also constructed eight test sets. Specifically, the first test set, ClinVar2, is built from new missense variants in the March 2023 release of ClinVar. After removing all variants that overlap with the training set, it contains a total of 31,071 valid variants. The second test set is the TP53 functional variant dataset[28], which includes 945 variants from TP53 mutation sites. ExoVar, PredictSNP, SwissVar, and VariBench constitute four established benchmark datasets for evaluating missense variant pathogenicity predictors[29]. Furthermore, for the task of predicting the pathogenicity of synonymous variants, we construct a dedicated test set by obtaining 826 synonymous variants from dbDSM[30] .

References

[1] Karczewski KJ, Francioli LC, Tiao G et al. The mutational constraint spectrum quantified from variation in 141,456 humans. Nature 2020;581(7809):434-443.

[2] Lek M, Karczewski KJ, Minikel EV et al. Analysis of protein-coding genetic variation in 60,706 humans. Nature 2016;536(7616):285-291.

[3] Bateman A, Coin L, Durbin R et al. The Pfam protein families database. Nucleic Acids Research 2004;32(Suppl 1):D138-D141.

[4] Hunter S, Apweiler R, Attwood TK et al. InterPro: the integrative protein signature database. Nucleic Acids Research 2009;37(Suppl 1):D211-D215.

[5] Fromentel CCD, Soussi T. Identification of discriminative gene-level and protein-level features associated with pathogenic gain-of-function and loss-of-function variants. American Journal of Human Genetics 2021;108(12):2301-2318.

[6] Piñero J, Bravo À, Queralt-Rosinach N et al. DisGeNET: a comprehensive platform integrating information on human disease-associated genes and variants. Nucleic Acids Research 2017;45(D1):D833-D839.

[7] Cooper GM, Stone EA, Asimenos G et al. Distribution and intensity of constraint in mammalian genomic sequence. Genome Research 2005;15(7):901-913.

[8] Hubisz MJ, Pollard KS, Siepel A. PHAST and RPHAST: phylogenetic analysis with space/time models. Briefings in Bioinformatics 2011;12(1):41-51.

[9] Pollard KS, Hubisz MJ, Rosenbloom KR et al. Detection of non-neutral substitution rates on mammalian phylogenies. Genome Research 2010;20(1):110-121.
[10] Rentzsch P, Witten D, Cooper GM et al. CADD: predicting the deleteriousness of variants throughout the human genome. Nucleic Acids Research 2019;47(D1):D886-D894.
[11] Quang D, Chen Y, Xie X. DANN: a deep learning approach for annotating the pathogenicity of genetic variants. Bioinformatics 2015;31(5):761-763.
[12] Shihab HA, Rogers MF, Gough J et al. An integrative approach to predicting the functional effects of non-coding and coding sequence variation. Bioinformatics 2015;31(10):1536-1543.
[13] Dong C, Wei P, Jian X et al. Comparison and integration of deleteriousness prediction methods for nonsynonymous SNVs in whole-exome sequencing studies. Human Molecular Genetics 2015;24(8):2125-2137.
[14] Qi H, Zhang H, Zhao Y et al. MVP predicts the pathogenicity of missense variants by deep learning. Nature Communications 2021;12(1):510.
[15] Ionita-Laza I, McCallum K, Xu B et al. A spectral approach integrating functional genomic annotations for coding and noncoding variants. Nature Genetics 2016;48(2):214-220.
[16] Li C, Zhi D, Wang K et al. MetaRNN: differentiating rare pathogenic and rare benign missense SNVs and indels using deep learning. Genome Medicine 2022;14(1):115.
[17] Adzhubei I, Jordan DM, Sunyaev SR. Predicting functional effect of human missense mutations using PolyPhen-2. Current Protocols in Human Genetics 2013;76(1):7.20.1-7.20.41.
[18] Schwarz JM, Cooper DN, Schuelke M et al. MutationTaster2: mutation prediction for the deep-sequencing age. Nature Methods 2014;11(4):361-362.
[19] Alirezaie N, Kernohan KD, Hartley T et al. ClinPred: prediction tool to identify disease-relevant nonsynonymous single-nucleotide variants. American Journal of Human Genetics 2018;103(4):474-483.
[20] Feng BJ. PERCH: a unified framework for disease gene prioritization. Human Mutation 2017;38(3):243-251.
[21] Carter H, Douville C, Stenson PD et al. Identifying Mendelian disease genes with the variant effect scoring tool. BMC Genomics 2013;14(Suppl 3):S3.
[22] Raimondi D, Tanyalcin I, Ferté J et al. DEOGEN2: prediction and interactive visualization of single amino acid variant deleteriousness in human proteins. Nucleic Acids Research 2017;45(W1):W201-W206.
[23] Choi Y, Sims GE, Murphy S et al. Predicting the functional effect of amino acid substitutions and indels. PLoS ONE 2012;7(10):e46688.
[24] Buske OJ, Manickaraj A, Mital S et al. Identification of deleterious synonymous variants in human genomes. Bioinformatics 2013;29(15):1843-1850.
[25] Gelfman S, Wang Q, McSweeney KM et al. Annotating pathogenic non-coding variants in genic regions. Nature Communications 2017;8(1):236.
[26] Cheng N, Li M, Zhao L et al. Comparison and integration of computational methods for deleterious synonymous mutation prediction. Briefings in Bioinformatics 2020;21(3):970-981.

[27] Landrum MJ, Lee JM, Benson M et al. ClinVar: improving access to variant interpretations and supporting evidence. Nucleic Acids Research 2018;46(D1):D1062-D1067.
[28] Fromentel CCD, Soussi T. TP53 tumor suppressor gene: a model for investigating human mutagenesis. Genes, Chromosomes and Cancer 1992;4(1):1-15.
[29] Grimm DG, Azencott CA, Aicheler F et al. The evaluation of tools used to predict the impact of missense variants is hindered by two types of circularity. Human Mutation 2015;36(5):513-523.
[30] Wen P, Xiao P, Xia J. dbDSM: a manually curated database for deleterious synonymous mutations. Bioinformatics 2016;32(12):1914-1916.

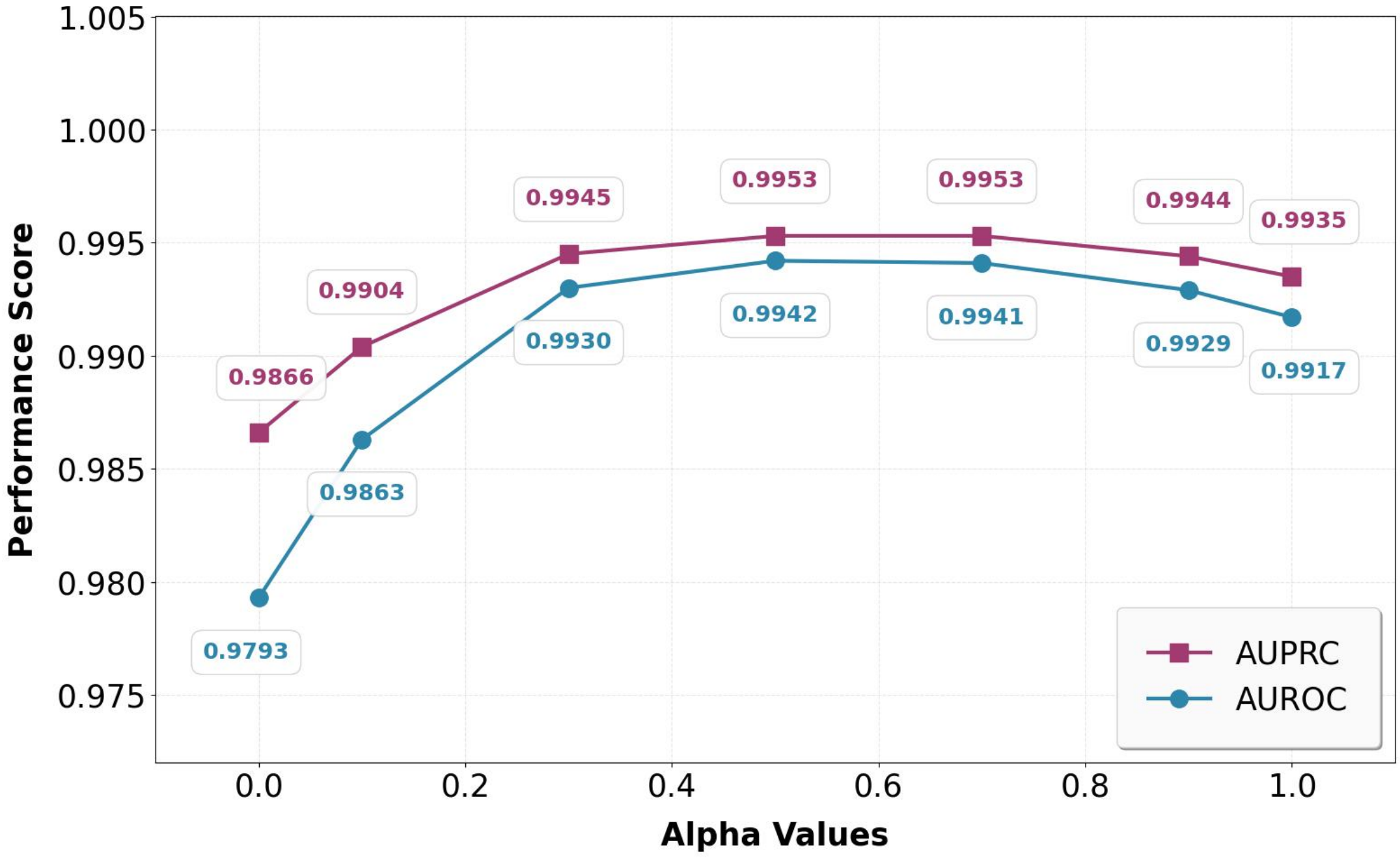

Figure S1: Parameter optimization for SIMLINK model.

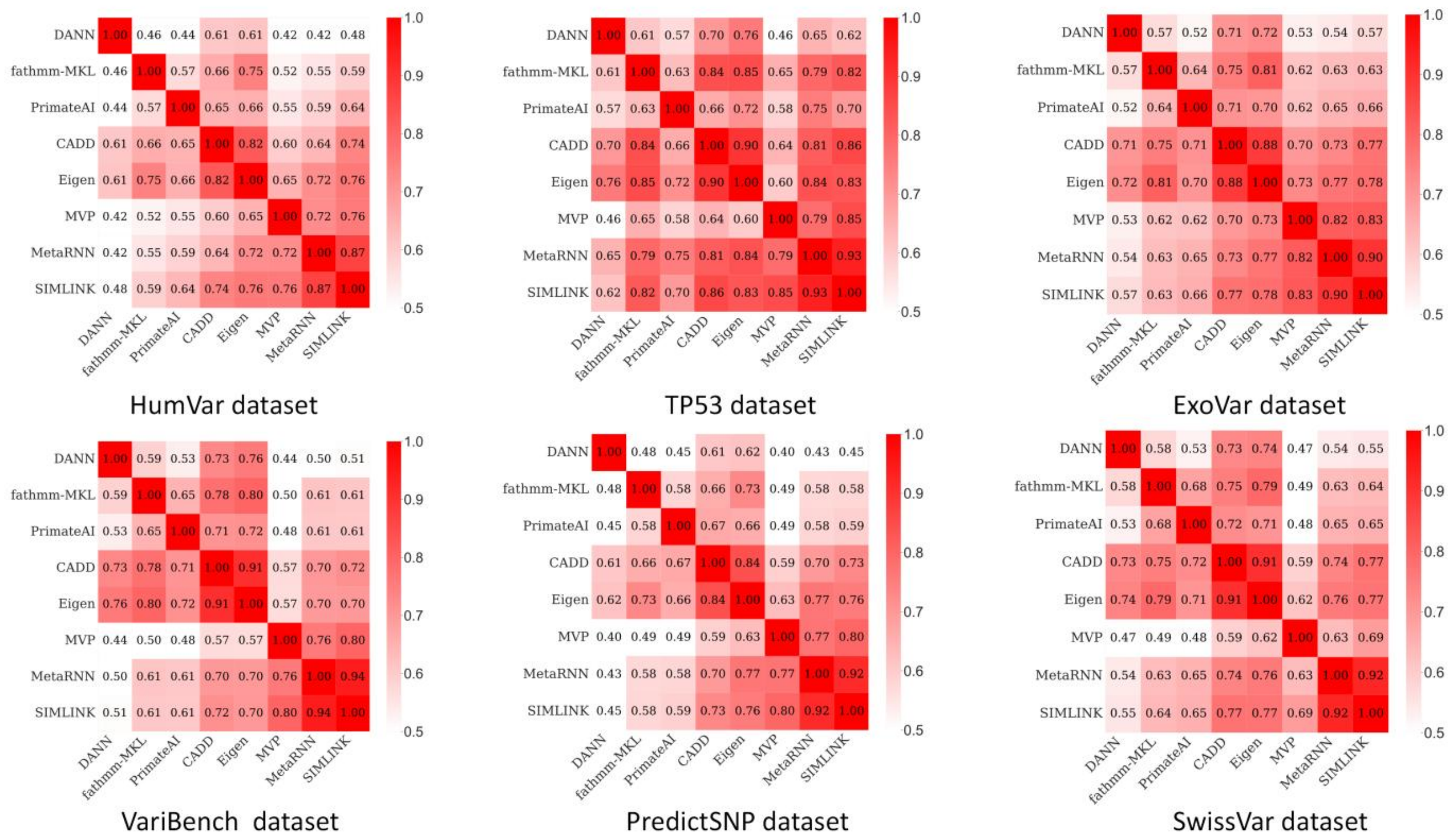

Figure S2: Consistency analysis of SIMLINK and other methods across six datasets.

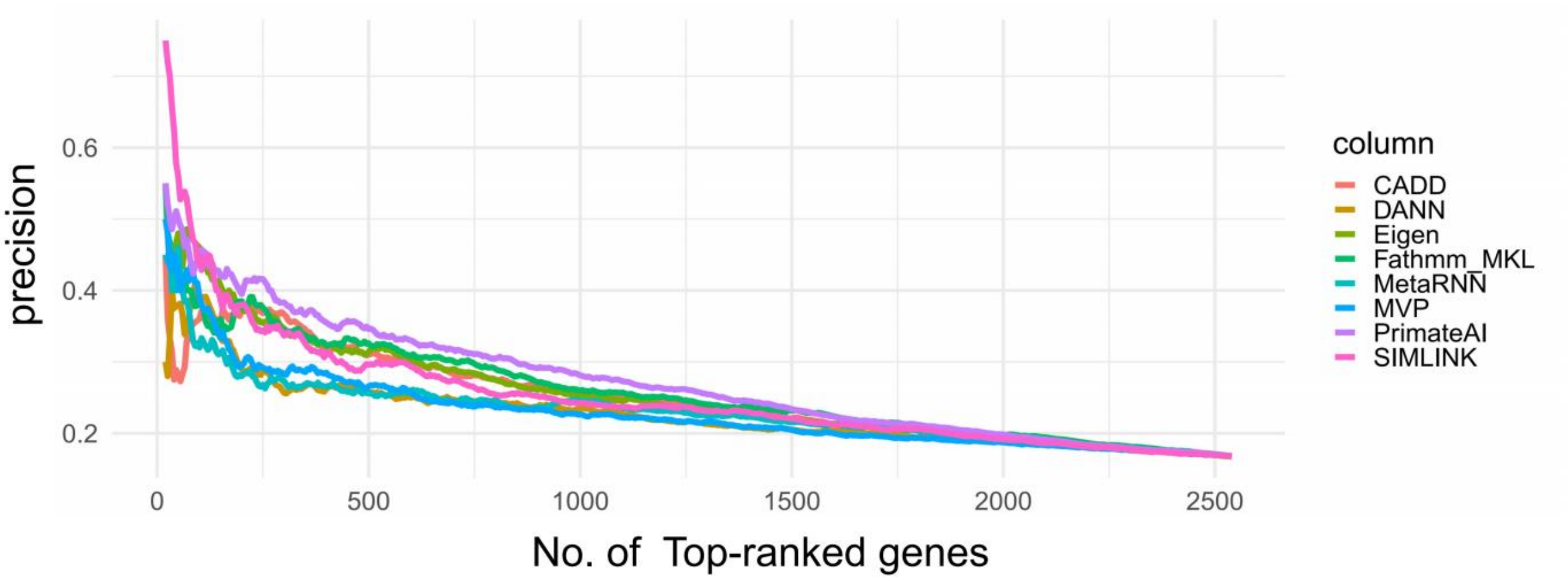

Figure S3: The precision of the top-ranked genes for each method.

Table S1：Performance evaluation under different α values across independent benchmark datasets.

Alpha	Humvar	ExoVar	VariBench	SwissVar	PredictSNP	ClinVar2	TP53	dbDSM
**0.0**	0.9866 ± 0.0000	0.9927 ± 0.0000	0.8090 ± 0.0000	0.7524 ± 0.0000	0.9620 ± 0.0000	0.9405 ± 0.0000	0.8360 ± 0.0000	0.6890 ± 0.0000
**0.1**	0.9901 ± 0.0015	0.9955 ± 0.0004	0.8247 ± 0.0077	0.7627 ± 0.0058	0.9677 ± 0.0025	0.9489 ± 0.0030	0.8508 ± 0.0002	0.6914 ± 0.0004
**0.3**	0.9940 ± 0.0029	0.9970 ± 0.0003	0.8481 ± 0.0096	0.7906 ± 0.0072	0.9713 ± 0.0031	0.9506 ± 0.0028	0.8492 ± 0.0007	0.6923 ± 0.0005
**0.5**	**0.9953 ± 0.0021**	**0.9974 ± 0.0001**	0.8656 ± 0.0097	0.7904 ± 0.0042	0.9787 ± 0.0023	**0.9511 ± 0.0009**	0.8436 ± 0.0008	0.6959 ± 0.0025
**0.7**	**0.9953 ± 0.0006**	0.9967 ± 0.0003	0.8841 ± 0.0018	**0.7956 ± 0.0009**	0.9815 ± 0.0029	0.9461 ± 0.0016	0.8354 ± 0.0011	0.6976 ± 0.0060
**0.9**	0.9949 ± 0.0004	0.9948 ± 0.0004	0.9084 ± 0.0071	0.7784 ± 0.0056	**0.9828 ± 0.0006**	0.9367 ± 0.0137	0.8230 ± 0.0016	**0.7095 ± 0.0135**
**1.0**	0.9935 ± 0.0001	0.9933 ± 0.0001	**0.9091 ± 0.0018**	0.7429 ± 0.0213	0.9809 ± 0.0001	0.9311 ± 0.0005	**0.8521 ± 0.0011**	0.6643 ± 0.0131
